# Correction to ‘The genome of *Cymbidium sinense* revealed the evolution of orchid traits’

**DOI:** 10.1111/pbi.70727

**Published:** 2026-07-16

Yang, F.‐X. , J. Gao , Y.‐L. Wei , et al. 2021. “The Genome of *Cymbidium sinense* Revealed the Evolution of Orchid Traits.” Plant Biotechnology Journal 19: 2501–2516. 10.1111/pbi.13676.34342129 PMC8633513

In the above article, the authors would like to update Figure 4b. The ultrastructure images of flower bud developmental stage 0 and stage 1 in the second row, which corresponded to plant morphology of stage 0 and stage 1 in the first row, were mistakenly displayed. The correct figure is shown below.

**FIGURE 4:**
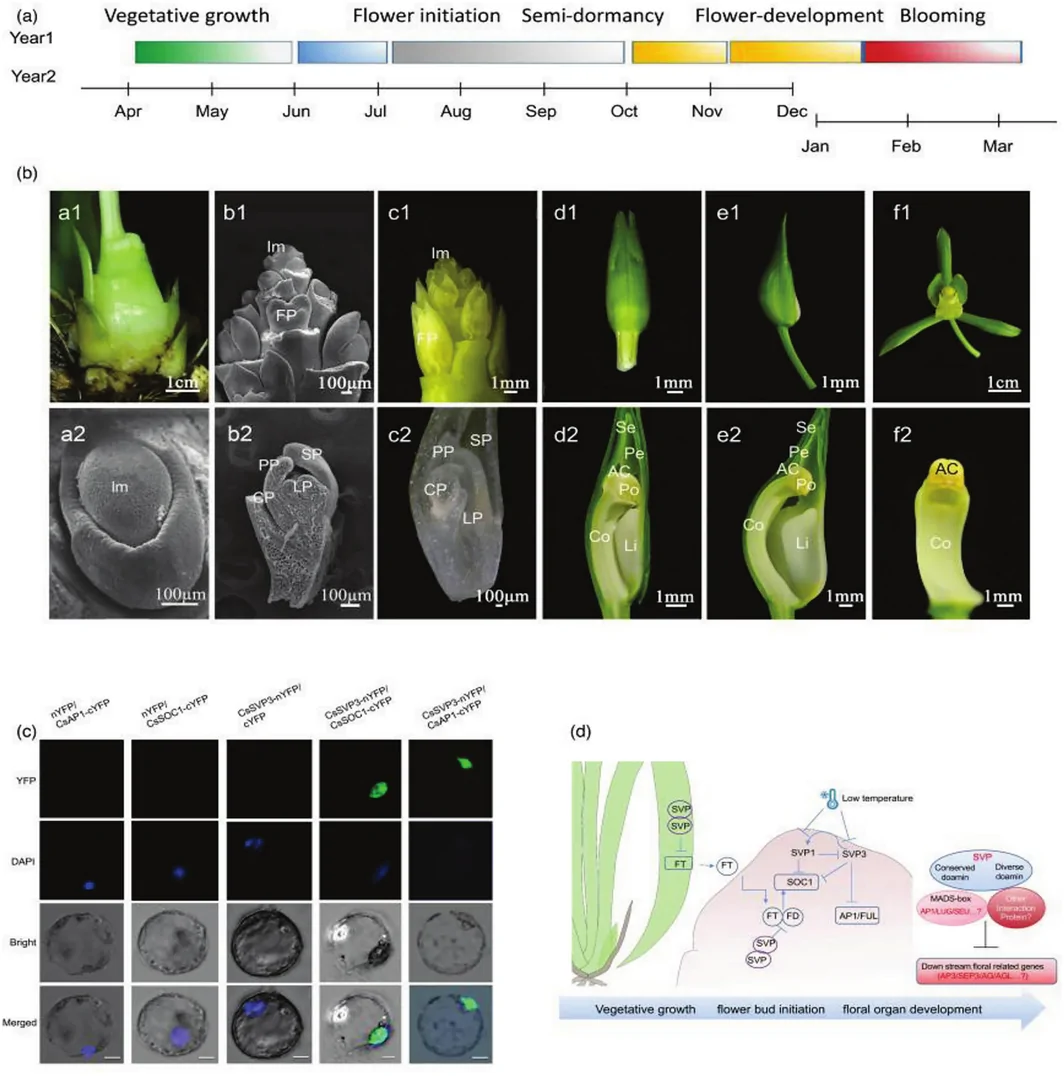
Seasonal flowering and the analysis of floral organ identity and flowering‐time‐related genes. (a) Schematic diagram of *C*. *sinense* seasonal growth and development in 12‐month cycle. (b) Floral development of *C*. *sinense*. (a–c) Scanning electron micrograph (SEM) of early floral developmental stages. (a) Dormant lateral buds (S0). (b) potential floral bud initiation in the lateral buds of developing shoots (S1). (c) Developing floret. d–f. developing flowers, Bar = 1 cm. The developing flower of stage 3 (d), stage 4 (e) and mature flowers (f). The first line: plant morphology, the second line: flower bud or floret form, the third line: microstructure of floral bud and floral organ. AC, anther cap; Br, Bract; Co, column; CP, column primordium; FP, floret primordium; Im, inflorescence meristem; Li, lip; Lo, locule; LP, labellum primordium; Pe, petal; Po, pollinium; PP, petal primordium; Se, sepal; SP, sepal primordium. (c) Biomolecular fluorescence complementation visualization. Fusion proteins CsSVP3‐YFPn and CsAP1‐YFPc or CsSOC1‐YFPc were co‐expressed in *C*. *sinense* protoplasts and YFP signals were detected in nuclei where the DAPI signal presented, while negative controls did not produce any BiFC fluorescence. (d) Control of flowering in *C*. *sinense*. Arrows with solid line indicated positive interaction, and right angle indicated negative interaction.

We apologize for these errors.

